# Supplementary material for: Biotic resistance predictably shifts microbial invasion regimes
Source: Nat Commun. 2025 Apr 27;16:3952. doi: 10.1038/s41467-025-59285-1 (PMC12034811; doi:10.1038/s41467-025-59285-1)
Supplement: Supplementary file 2 — Description of Additional Supplementary Files [file 41467_2025_59285_MOESM2_ESM.pdf]

Description of Additional Supplementary Files

Supplementary Dataset 1

`./rawdata_experiment/` contains code and data files to reproduce all figures about experimental results (including predictions). Run `figs_experiment.ipynb` for plotting the figures. Data files for each figure are listed below.

* Fig.1c - SL_comp_cfu.xlsx

* Fig.1d - CM_comp_cfu.xlsx

* Fig.2c,d - SL_inv_OD.xlsx

* Fig.2e - CM_inv_luc.xlsx

* Fig.3b - SL_comp_cfu.xlsx (i, ii, iii), SL_inv_OD.xlsx (iii)

* Fig.3c - CM_comp_cfu.xlsx (i, ii, iii), CM_inv_luc.xlsx (iii)

* Sup Fig.1a - SL_pH.xlsx

* Sup Fig.1b,c - SL_growth_diffpH.csv (the growth curve, with a "Sample Layout" section within the file), SL_growth_diffpH_inipH.csv (the initial pH of the media)

* Sup Fig.2b - CM_comm_stability.xlsx

* Sup Fig.2c - CM_pH.xlsx

* Sup Fig.2d - CM_OD.csv

* Sup Fig.3 - SL_comp_cfu.xlsx

* Sup Fig.4 - CM_comp_cfu.xlsx

* Sup Fig.5 - SL_mig_CFU_timeseries_40mM.xlsx

* Sup Fig.6 - SL_inv_OD.xlsx

* Sup Fig.7 - CM_inv_luc.xlsx

* Sup Fig.8 - SL_comp_cfu.xlsx

* Sup Fig.9 - CM_comp_cfu.xlsx

Note: for the sequencing and the simulated results as below, the raw data are available only in [Figshare](https://doi.org/10.6084/m9.figshare.27168834) due to large size

`./raw_16S_sequencing/` contains raw Illumina sequencing files and the bash script for generating the read count table in `./rawdata_experiment/`.

`./rawdata_simulations/` contains code and data files to reproduce all figures about simulation results. Run `figs_simulations.ipynb` for plotting the figures. Processed data are available in `pickle` format in `./cr/` for Consumer-resource models and `./lv/` for Lotka-Volterra models. The raw simulation data are not necessary for reproducing the figures and are not provided due to their large size, but could be locally generated by running first `main_simulations.py` then `analysis.py`. See `./rawdata_simulations/readme_simulations.md` for details.

* Fig. 4

* Sup Fig.10-19
