# Supplementary material for: Biotic resistance predictably shifts microbial invasion regimes
Source: Nat Commun. 2025 Apr 27;16:3952. doi: 10.1038/s41467-025-59285-1 (PMC12034811; doi:10.1038/s41467-025-59285-1)
Supplement: Supplementary file 4 — Reporting Summary [file 41467_2025_59285_MOESM4_ESM.pdf]

nature portfolio

Corresponding author(s): Christoph Ratzke

Last updated by author(s): Dec 13, 2024

Reporting Summary

Nature Portfolio wishes to improve the reproducibility of the work that we publish. This form provides structure for consistency and transparency in reporting. For further information on Nature Portfolio policies, see our Editorial Policies and the Editorial Policy Checklist.

Statistics

For all statistical analyses, confirm that the following items are present in the figure legend, table legend, main text, or Methods section.

n/a	Confirmed	
☐	☒	The exact sample size (*n*) for each experimental group/condition, given as a discrete number and unit of measurement
☐	☒	A statement on whether measurements were taken from distinct samples or whether the same sample was measured repeatedly
☐	☒	The statistical test(s) used AND whether they are one- or two-sided<br>*Only common tests should be described solely by name; describe more complex techniques in the Methods section.*
☒	☐	A description of all covariates tested
☒	☐	A description of any assumptions or corrections, such as tests of normality and adjustment for multiple comparisons
☐	☒	A full description of the statistical parameters including central tendency (e.g. means) or other basic estimates (e.g. regression coefficient) AND variation (e.g. standard deviation) or associated estimates of uncertainty (e.g. confidence intervals)
☒	☐	For null hypothesis testing, the test statistic (e.g. *F*, *t*, *r*) with confidence intervals, effect sizes, degrees of freedom and *P* value noted<br>*Give P values as exact values whenever suitable.*
☒	☐	For Bayesian analysis, information on the choice of priors and Markov chain Monte Carlo settings
☒	☐	For hierarchical and complex designs, identification of the appropriate level for tests and full reporting of outcomes
☒	☐	Estimates of effect sizes (e.g. Cohen's *d*, Pearson's *r*), indicating how they were calculated

Our web collection on statistics for biologists contains articles on many of the points above.

Software and code

Policy information about availability of computer code

Data collection	BMG FLUOstar and accompanying software was used to collect OD600nm and bioluminescence data.<br>Python version 3.11 and scipy package version 1.13.0 was used for simulations.
Data analysis	Python version 3.11 was used for data analysis.

For manuscripts utilizing custom algorithms or software that are central to the research but not yet described in published literature, software must be made available to editors and reviewers. We strongly encourage code deposition in a community repository (e.g. GitHub). See the Nature Portfolio guidelines for submitting code & software for further information.

Data

Policy information about availability of data

All manuscripts must include a data availability statement. This statement should provide the following information, where applicable:

- Accession codes, unique identifiers, or web links for publicly available datasets
- A description of any restrictions on data availability
- For clinical datasets or third party data, please ensure that the statement adheres to our policy

The experimental and simulation data generated in this study have been deposited on Figshare (https://doi.org/10.6084/m9.figshare.27168834). The experimental data are also available in the Source Data File.

Research involving human participants, their data, or biological material

Policy information about studies with human participants or human data. See also policy information about sex, gender (identity/presentation), and sexual orientation and race, ethnicity and racism.

Reporting on sex and gender	n/a
Reporting on race, ethnicity, or other socially relevant groupings	n/a
Population characteristics	n/a
Recruitment	n/a
Ethics oversight	n/a

Note that full information on the approval of the study protocol must also be provided in the manuscript.

Field-specific reporting

Please select the one below that is the best fit for your research. If you are not sure, read the appropriate sections before making your selection.

☒ Life sciences ☐ Behavioural & social sciences ☐ Ecological, evolutionary & environmental sciences

For a reference copy of the document with all sections, see nature.com/documents/nr-reporting-summary-flat.pdf

Life sciences study design

All studies must disclose on these points even when the disclosure is negative.

Sample size	For each pair of invader and residents, invasion experiments were carried across 12 wells, under 8 dispersal rates, and over 10 days. 2 different study systems (two-strain and multi-strain) each with 4 conditions with varying biotic resistance levels were enough to represent widely different biotic resistance scenarios, while remaining experimentally feasible.
Data exclusions	One replicate in invasion experiment for multi-strain system was excluded due to massive cross-contamination, likely caused by broken bubbles when handling the cultures using the tip-based liquid handling system. A few points in interaction measurements or invasion experiment were excluded due to cross-contamination or liquid transfer error.
Replication	All experiments were replicated at least 3 times. All replicates were successful for interaction measurements for both systems and invasion experiments for the two-strain system. The results shown in Supplementary Fig. 5 were from an independent replicate with slightly modified protocol to incorporate daily plating and colony counting, therefore, the invasion speeds were not included in main text Fig. 3b(iii) even though the values agreed very well with the other replicates. One replicate in invasion experiment for multi-strain system failed due to massive cross-contamination as described above; because of its minor contribution in supporting the main message and the high cost and time requirement, no new replicate was added to compensate for the failed one.
Randomization	The experiments were not randomized.
Blinding	Blinding was not necessary for the experiments. The invasion outcome was tracked by OD600nm, bioluminescence, or colony morphologies, all of which were objective measurements that would not change at the whim of the individual.

Reporting for specific materials, systems and methods

We require information from authors about some types of materials, experimental systems and methods used in many studies. Here, indicate whether each material, system or method listed is relevant to your study. If you are not sure if a list item applies to your research, read the appropriate section before selecting a response.

Materials & experimental systems

n/a	Involved in the study
☒	☐ Antibodies
☒	☐ Eukaryotic cell lines
☒	☐ Palaeontology and archaeology
☐	☒ Animals and other organisms
☒	☐ Clinical data
☒	☐ Dual use research of concern
☒	☐ Plants

Methods

n/a	Involved in the study
☒	☐ ChIP-seq
☒	☐ Flow cytometry
☒	☐ MRI-based neuroimaging

Animals and other research organisms

Policy information about studies involving animals; ARRIVE guidelines recommended for reporting animal research, and Sex and Gender in Research

Laboratory animals

Lab bacteria strains were used in this study.
In the two-strain system, Sporosarcina ureae (Su) was from Ward's Science (#470179-156), and Lactiplantibacillus plantarum (Lp) was from ATCC8014. In the multi-strain system, the invader Pseudomonas aeruginosa (Pa) is a PA01 strain which we genetically tagged with luciferase (see Methods). The 16 strains that we used to build resident communities (Supplementary Fig. 2) were previously isolated from the gut of Caenorhabditis elegans (C. elegans) from Northern Germany1 (MYb27) or Massachusetts, United States2 (the other 15 strains).
1. Dirksen, P. et al. The native microbiome of the nematode Caenorhabditis elegans: gateway to a new host-microbiome model. BMC Biol. 14, 38 (2016).
2. Ortiz, A., Vega, N. M., Ratzke, C. & Gore, J. Interspecies bacterial competition regulates community assembly in the C. elegans intestine. ISME J. 15, 2131–2145 (2021).

Wild animals

n/a

Reporting on sex

n/a

Field-collected samples

n/a

Ethics oversight

n/a

Note that full information on the approval of the study protocol must also be provided in the manuscript.

Plants

Seed stocks

n/a

Novel plant genotypes

n/a

Authentication

n/a
